# Evaluation of a New Paracingulate Sulcus Identification and Measurement Protocol

**DOI:** 10.1002/hbm.70574

**Published:** 2026-06-15

**Authors:** Héloïse de Vareilles, Samantha C. Mitchell, Jane R. Garrison, Shun‐Chin Jim Wu, Laura Alvarez‐Sanchez, Reuben Thomas, Suveththa Kugan, Atheer Al‐Manea, Michail Mamalakis, Lynn Egeland Mørch‐Johnsen, Ingrid Agartz, John Suckling, Jon S. Simons, Graham K. Murray

**Affiliations:** ^1^ Department of Psychiatry University of Cambridge Cambridge UK; ^2^ Department of Psychology University of Cambridge Cambridge UK; ^3^ Department of Computer Sciences and Technology University of Cambridge Cambridge UK; ^4^ Norment, Division of Mental Health and Addiction Oslo University Hospital, Institute of Clinical Medicine, University of Oslo Oslo Norway; ^5^ Department of Psychiatry Østfold Hospital Grålum Norway; ^6^ Department of Clinical Research Østfold Hospital Grålum Norway; ^7^ Department of Psychiatric Research Diakonhjemmet Hospital Oslo Norway; ^8^ Cambridgeshire and Peterborough NHS Foundation Trust Cambridge UK

## Abstract

The folding pattern of the anterior cingulate cortex is variable among individuals. The paracingulate sulcus (PCS), in particular, can be difficult to identify because of this variability. In this paper, we assessed the benefits of a new protocol to identify the PCS and measure its length using 3D reconstructions of the brain obtained through the BrainVISA software. Both the previous state‐of‐the‐art protocol and the new protocol were applied to identify the PCS in subjects from a UK cohort (*n* = 50), with the length of the PCS computed automatically by BrainVISA after PCS identification. We assessed inter‐rater reliability for PCS length under both protocols and the correlation of PCS length values output by the two protocols. Inspection of the results indicated an advantage of the new protocol as there are 8 out of 100 hemispheres in which the old protocol detects a PCS due to the mislabelling of the intra‐limbic sulcus as the cingulate sulcus and the new protocol does not. This advantage is conferred by the incorporation of identification of the intra‐limbic sulcus (when present) in the new protocol. For instances where the protocols agreed on the presence of a PCS, the new protocol for PCS length obtained intra‐class correlations of 0.85 and 0.86 on respectively untrained and trained experimenters, compared to 0.81 when trained experimenters used the previous protocol. The PCS length correlation between both protocols was 0.73 for the entire sample and 0.85 after excluding instances where the protocols disagreed on the presence of a PCS. These findings suggest that taking into account the intra‐limbic sulcus and taking advantage of 3D sulcal visualisation may help improve reliable PCS identification, and that the new protocol is a reliable tool that is likely to prove useful in research into cingulate and paracingulate cortical folding.

## Graphical abstract

The paracingulate sulcus (PCS) can be difficult to identify due to its variability. A new protocol is proposed to identify the PCS and measure its length using 3D reconstructions of the brain. The protocol aims to improve the anatomical accuracy and reduce the ambiguity of PCS identifications and demonstrates good inter‐rater reliability.

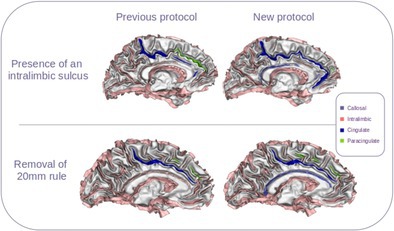

## 1 Introduction

The way the brain folds is mostly determined in utero during a critical period for neurodevelopment, such that the pattern of brain folding remains stable later in life, as demonstrated in the anterior cingulate cortex (Cachia et al. 2016). Individual differences in brain folding pattern are related to differences in brain functional connectivity (Fedeli et al. 2020) and to cognitive and functional outcomes (Mangin et al. 2010). Therefore, investigating the presence and pattern of sulci (brain folds) can be informative both on brain function and on neurodevelopmental events (Cachia et al. 2021), and may be of importance for target selection in brain stimulation research and clinical practice. In this regard, the sulcation pattern within the medial prefrontal and cingulate cortices is a subject of interest in the context of the cognitive processes such as reality monitoring (Simons et al. 2017; Perret et al. 2021) and psychiatric disorders such as schizophrenia (e.g., Garrison et al. 2015; Garrison et al. 2018; Rollins et al. 2020; Meredith et al. 2012; Ćurčić‐Blake et al. 2023).

The paracingulate sulcus (PCS) is a tertiary‐like fold, within the medial prefrontal cortex, lying dorsal to the cingulate sulcus and is specific to humans and great apes (Amiez et al. 2021; Miller et al. 2021). As documented in seminal papers by Paul and colleagues (Paus, Tomaiuolo, et al. 1996; Paus, Otaky, et al. 1996), the PCS shows considerable variability between individuals, and even between hemispheres, can be absent in either or both hemispheres and, when present, often exhibits variable, fragmented configurations. The configuration of the PCS has been reported to relate to function in cognitive processes (reality monitoring: Buda et al. 2011; Simons et al. 2017; executive function and cognitive control: Cachia et al. 2014, 2017) and seems to play a role in some pathologies, such as schizophrenia (e.g., Meredith et al. 2012; Garrison et al. 2015; Rollins et al. 2020; Ćurčić‐Blake et al. 2023; Wu et al. 2025), bipolar disorder (Nenadic et al. 2015; Sarrazin et al. 2018; Wu et al. 2025), Parkinson's disease (Karagoz et al. 2025), and frontotemporal dementia (Harper et al. 2024).

While the presence, morphology and importance of the PCS have been investigated between different clinical or demographic subgroups, the studies often yielded contradictory results. For example, Paus, Tomaiuolo, et al. (1996) found that women tended to have either more prominent or absent PCS, while men had more of the intermediate category of “present PCS”, while further studies reported men to have longer PCS than women (right hemisphere: Leonard et al. 2009; both hemispheres: Clark et al. 2010). Conflicting results emanate about asymmetry, with some studies indicating men, but not women, showing more leftward asymmetry (Yücel et al. 2001; Whittle et al. 2009), and others finding the opposite (Leonard et al. 2009; Clark et al. 2010). Possible explanations for these previous heterogeneous results could relate to discrepancies in the definition of the PCS. The aforementioned inter‐hemisphere and inter‐individual variability in the presence and configuration of the PCS means that identification of the structure can be ambiguous in some individuals. In addition, the definition of the PCS relies on the identification and orientation of the cingulate sulcus, which can sometimes be ambiguous itself depending on the sulcal configuration of a given hemisphere.

As a result, a number of protocols have been developed to define the PCS and investigate functional or pathological outcomes which could be related to its configuration. The two most widely used protocols either result in a classification of the PCS as prominent, present or absent (Yücel et al. 2001) or in a delineation of the PCS allowing for a continuous length metric to be extracted (Garrison 2017; Garrison et al. 2018). In both cases, the PCS is defined as a sulcus parallel to the cingulate sulcus, starting anteriorly from the point where it takes an antero‐posterior orientation and stopping posteriorly after a virtual line stemming from the anterior commissure and perpendicular to the virtual line crossing both the anterior and posterior commissures (VAC line). These two previous protocols involve either qualitative or quantitative assessment of sulcal length based on 2D MRI slices. This is not optimal to assess features that are relevant to accurate and reliable identification of sulci in this region, such as sulcal depth.

New tools allow for 3D reconstruction of the brain and 3D extraction of sulci (Rivière et al. 2022, https://brainvisa.info/), which enable researchers to identify the PCS with more anatomical consistency. Notably, the cingulate sulcus, adjacent to the PCS, is a primary fold and as such is expected to be a particularly deep sulcus within the medial surface (Dubois et al. 2008). Therefore, being able to inform the decision on cingulate sulcus identification not only in terms of position (dorsal to the callosal sulcus) but also in terms of depth relative to neighbouring sulci can remove ambiguity in the identification of the PCS, highlighting the benefits of a revision of the above‐mentioned protocols.

In this context, we have developed a new protocol for the identification of the PCS (Mitchell et al. 2023) described in Supporting Information S1. This allows researchers to identify the PCS on 3D brain reconstructions, opening possibilities for sulcal studies going beyond solely the length of the sulcus (e.g., automatic extraction of depth or width, or extraction of 3D object for more comprehensive pattern studies). Another key aspect of the new protocol is how it manages the case of an interrupted paracingulate sulcus. In previous protocols, sulcal elements were excluded from the PCS according to a criterion based on the length of interruption, which led similar sulcus elements to be either included or excluded from the PCS based on neighbouring configurations. The new protocol also takes into account the presence of an intra‐limbic sulcus, an uncommon variant of a shallow sulcus superior to the callosal sulcus and inferior to the cingulate sulcus (Paus, Tomaiuolo, et al. 1996). When an intra‐limbic sulcus is present, the new protocol labels it as such, and the sulcus above it is labelled as cingulate; in contrast, the previous protocol may have erroneously identified an intra‐limbic sulcus as cingulate and labelled the sulcus above it as paracingulate. This new protocol therefore focuses on positional and orientational parameters to identify the PCS, after a rigorous definition of the cingulate sulcus inferred from developmental considerations.

The current study aims to compare the new protocol (Mitchell et al. 2023), hereafter referred to as “new protocol”, to the current state‐of‐the‐art protocol (Garrison 2017), hereafter referred to as the “previous protocol”, in terms of misclassifications, similarity of output metrics and inter‐rater reliability.

## 2 Materials and Methods

### 2.1 Subjects

The current study involved sampling from the BeneMin cohort (Deakin et al. 2018), composed of patients in a first episode of schizophrenia, schizophreniform, or schizoaffective psychosis, imaged in different scanning centres in the United Kingdom. 50 random subjects were selected from this cohort to assess the reliability of the newly proposed protocol and its relationship with the previous one. A summary of their clinical information is presented in Table 1.

**TABLE 1:** Clinical data on the sample.

Characteristics (number of subjects)	Mean ± standard deviation (range) or *N* (percentage)
Age (*n* = 49)	25.1 ± 4.9 (17–37)
Sex (*n* = 50)	
Female	13 (26%)
Male	37 (74%)
PANSS score (*n* = 43)	
Negative subscale score	18.7 (5.4)
Positive subscale score	16.9 (5.2)
Total PANSS score	34.2 (7.0)
CDSS score (*n* = 38)	12.4 (7.1)
GAF score (*n* = 50)	55.6 (9.2)
Body‐mass index (*n* = 48)	28.6 (7.7)
Current IQ (*n* = 49)	90.8 (15.2)
Pre‐morbid IQ (*n* = 47)	98.9 (11.6)

Abbreviations: CDSS: Calgary Depression Scale for Schizophrenia; GAF: Global Assessment of Functioning; IQ: Intelligence Quotient; PANSS: Positive and Negative Symptom Scale.

### 2.2 Image Preprocessing

**FIGURE 1:**
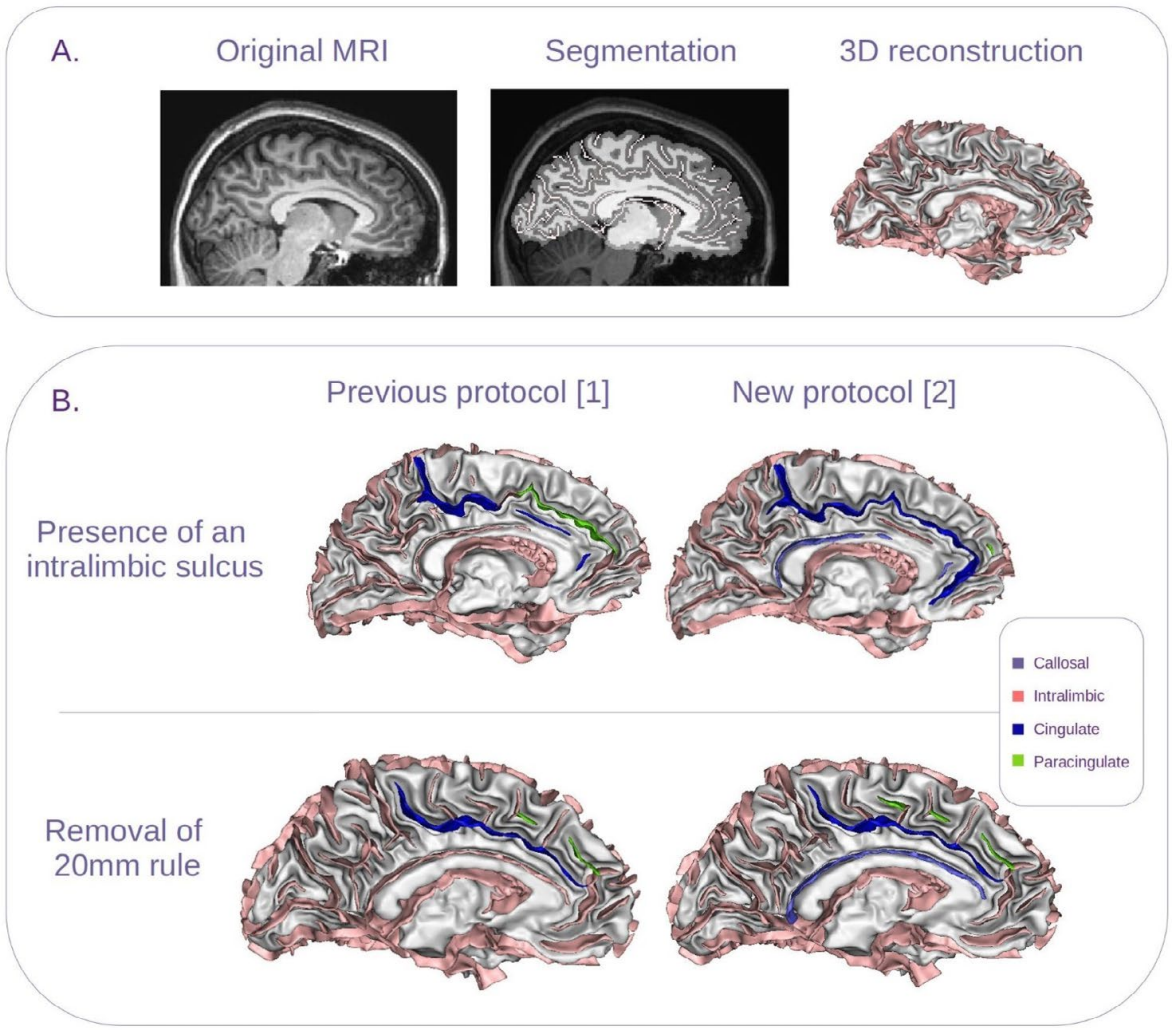
Illustration of brain reconstruction and PCS labelling. (A) Illustration of the BrainVISA 3D reconstruction, containing the grey‐white matter interface and sulcal reconstructions. (B) Illustration of two of the discrepancies in labelling between the previous ([1] Garrison 2017) and new ([2] Mitchell et al. 2023) protocols. Top row: Subject with an intra‐limbic sulcus, bottom row: Subject with a total compound gap between PCS elements of more than 20 mm. Left: Possible labels according to the previous protocol, right: Labels according to the new protocol. Note that the previous protocol would suggest looking into neighbouring slices to take a decision, so the label indicated here for the intra‐limbic case could be disputed. Also note that the previous protocol does not guide through the labelling of sulci other than the cingulate and PCS, while the new protocol guides through a general identification of sulci in order to identify the PCS. Light pink: Unlabelled, purple: Callosal sulcus, orange: Intra‐limbic sulcus, dark blue: Cingulate sulcus, green: Paracingulate sulcus (PCS).

The study used T1 weighted magnetic resonance images (MRI) acquired at 3 T (BeneMin, coordinated sequences from multiple centres, Deakin et al. 2018). Scans were processed using the BrainVISA processing pipeline (version 5.0.4; Rivière et al. 2022): the images are first segmented between grey matter, white matter, and cerebrospinal fluid. The hemispheres are split, and a brain hull is computed by morphological closing. Along with other objects, 3D representations of the grey‐white matter interface and ribbon‐like objects representing the sulci are constructed. The sulcal objects are constructed as the median surface within the cerebrospinal fluid between two gyral walls, constrained on the bottom by the grey matter surface, and on the top by the brain hull. A 3D reconstruction of the grey‐white matter interface is generated along the sulcal objects for visualisation, as represented in Figure 1A.

### 2.3 Protocols and Manual Identification

The protocols compared in this study are the two protocols resulting in quantitative length of the PCS (Garrison 2017; Mitchell et al. 2023). The previous protocol (Garrison 2017) relies on the MRI slice 4 mm deep inside the medial surface: the first major sulcus above the callosal sulcus is defined as the cingulate sulcus and the PCS is defined as the second sulcus dorsal to the callosal sulcus. The PCS is then delineated based on criteria following the seminal paracingulate classification protocol (Yücel et al. 2001), in which the PCS is qualified as prominent if it is either more than 40 mm long or has less than 20 mm interruption within its course. Therefore, in the previous protocol (Garrison 2017) the delineation starts anteriorly when the sulcus takes an antero‐posterior orientation, and the labelling stops either when the VAC line is met or when a total of 20 mm gap within the sulcus is reached.

The new protocol (Mitchell et al. 2023) is a revision of the previous one, accounting for the additional information gained through 3D visualisation and amending several anatomical considerations. Specifically, the main differences in the new protocol are the following: (1) the sulci are labelled throughout most of the cingulate region to prevent potential confusion between neighbouring sulci, (2) the depth of sulci was introduced as a criterion for decision‐making, (3) the potential presence of an intra‐limbic sulcus between the callosal sulcus and the cingulate sulcus was acknowledged (as reported in Leonard et al. 2009), and (4) the previous protocol discarded sulcal elements matching the PCS when interruptions exceeded 20 mm; this rule was not applied in the new protocol. The main differences between the protocols are illustrated on Figure 1B.

On the 50 subjects from the BeneMin cohort, manual identification of the PCS was undertaken by two different experimenters (HV and SM) using these two versions of the protocol. Since these experimenters developed the new protocol (HV and SM) and were therefore specifically trained on it, two other experimenters (RT and SK), blind to the new protocol elaboration, were asked to label the hemispheres without training, strictly following the new protocol.

### 2.4 Comparison of Previous and New Labelling Protocols

Six different sets of labels were obtained on the BeneMin subjects by different experimenters, as detailed in Table 2: (1) PREVIOUS1—HV using the previous protocol, (2) PREVIOUS2—SM using the previous protocol, (3) NEW1—HV using the new protocol, (4) NEW2—SM using the new protocol, (5) NEW3—RT using the new protocol, (6) NEW4—SK using the new protocol. The length of the PCS was automatically extracted (at the brain hull junction) and used as a continuous variable. A seventh set of labels was obtained from an untrained experimenter but discarded after visual inspection for not following the protocol.

**TABLE 2:** Summary of set of labels obtained, detailing protocol used and experimenter.

Set of labels	Protocol		Experimenter
	Garrison (2017)	Mitchell et al. (2023)	
PREVIOUS1	✓		HV
PREVIOUS2	✓		SM
NEW1		✓	HV
NEW2		✓	SM
NEW3		✓	RT
NEW4		✓	SK

To assess the reliability of the protocols, two‐way mixed‐effect model intraclass correlation coefficients (ICC) were computed between experimenters of each training level using the same protocol. Hence, three ICCs were computed: one between PREVIOUS1 and PREVIOUS2 (ICCprevious), one between NEW1 and NEW2 (ICCnew), and one between NEW3 and NEW4 (ICCnew_blind).

To aid statistical validity of the ICC, any values of 0 cm (absent PCS) for each pair of labels were removed (resulting in *n* = 82 for ICCprevious, *n* = 77 for ICCnew, and *n* = 80 for ICCnew_blind). Specifically, if a hemisphere was deemed to be absent of any PCS (length = 0 cm) by at least one experimenter, it was excluded. The remaining data were then transformed to have a normal distribution through a square root transformation, as verified by the Shapiro–Wilk test.

The ICCs obtained were also reported with the untransformed data, including absent PCS (length = 0 cm). In this case, the confidence intervals and *p*‐values were not reported as this data could not be transformed to a normal distribution.

To assess how the PCS measures compared between the two protocols, the lengths obtained from PREVIOUS1 and PREVIOUS2 were averaged (mean(PREVIOUS1, PREVIOUS2)), as well as the lengths from NEW1 and NEW2 (mean(NEW1, NEW2)). A Spearman correlation coefficient was computed between the resulting averages, after removing the subjects with an absent PCS according to either protocol, to prevent multiple subjects having the same rank, resulting in *n* = 76. Another Spearman correlation was also computed for all individuals combined, as a summary indicator of methodological agreement; we reasoned this computation may be of value to some scientists seeking a single summary metric of how the methodological choice will impact the output measurements.

For an additional assessment of agreement between raters and the differences induced by the two protocols on PCS measures when the PCS is present according to both protocols, we applied the method recommended by Bland and Altman for repeated measurements (Martin Bland and Altman 1986). This method is designed to assess whether a new protocol's outputs agree sufficiently with a previous one for the new protocol to become the new standard, using graphical methods and accessible calculations, rather than correlations that can lead to misleading interpretations. These analyses were conducted on those hemispheres where the PCS was deemed to be present by both raters (HV and SM) using both protocols. Using the length obtained with the previous protocol, we plotted the difference in length between experimenters (HV and SM) compared to the mean value and computed the mean difference and standard deviation of these differences. We then repeated this using the new protocol. Finally, taking as input the mean values obtained with the previous protocol, and those obtained with the new protocol, we plotted their differences versus their mean, and added a standard deviation of the difference between the means corrected for multiple measurements:

$${\mathrm{std}}_{\text{corrected}}=\sqrt{{\mathrm{std}}_{\text{mean}}^{2}+\frac{1}{4}{\mathrm{std}}_{\text{prev}}^{2}+\frac{1}{4}{\mathrm{std}}_{\mathrm{new}}^{2}}$$

With *std*_*corrected*_ the standard deviation corrected for multiple measurements, *std*_*mean*_ the standard deviation of the difference between the means for each method, *std*_*prev*_ the standard deviation of the differences between experimenters using the previous protocol, and *std*_*new*_ the standard deviation of the differences between experimenters using the new protocol.

We combined left and right hemispheres for our analyses to produce clear, easily reportable metrics of assessing each protocol on brain imaging data. However, in doing so, we combined data from both hemispheres of each participant, violating non‐independence assumptions of the statistical methods used. To help address whether this violation affected the results, we also conducted separate analyses for each hemisphere (results reported in the Supporting Information S1).

## 3 Results

### 3.1 Assessment of Previous and New Labelling Protocols

#### 3.1.1 Inter‐Rater Consistency in Labelling

**FIGURE 2:**
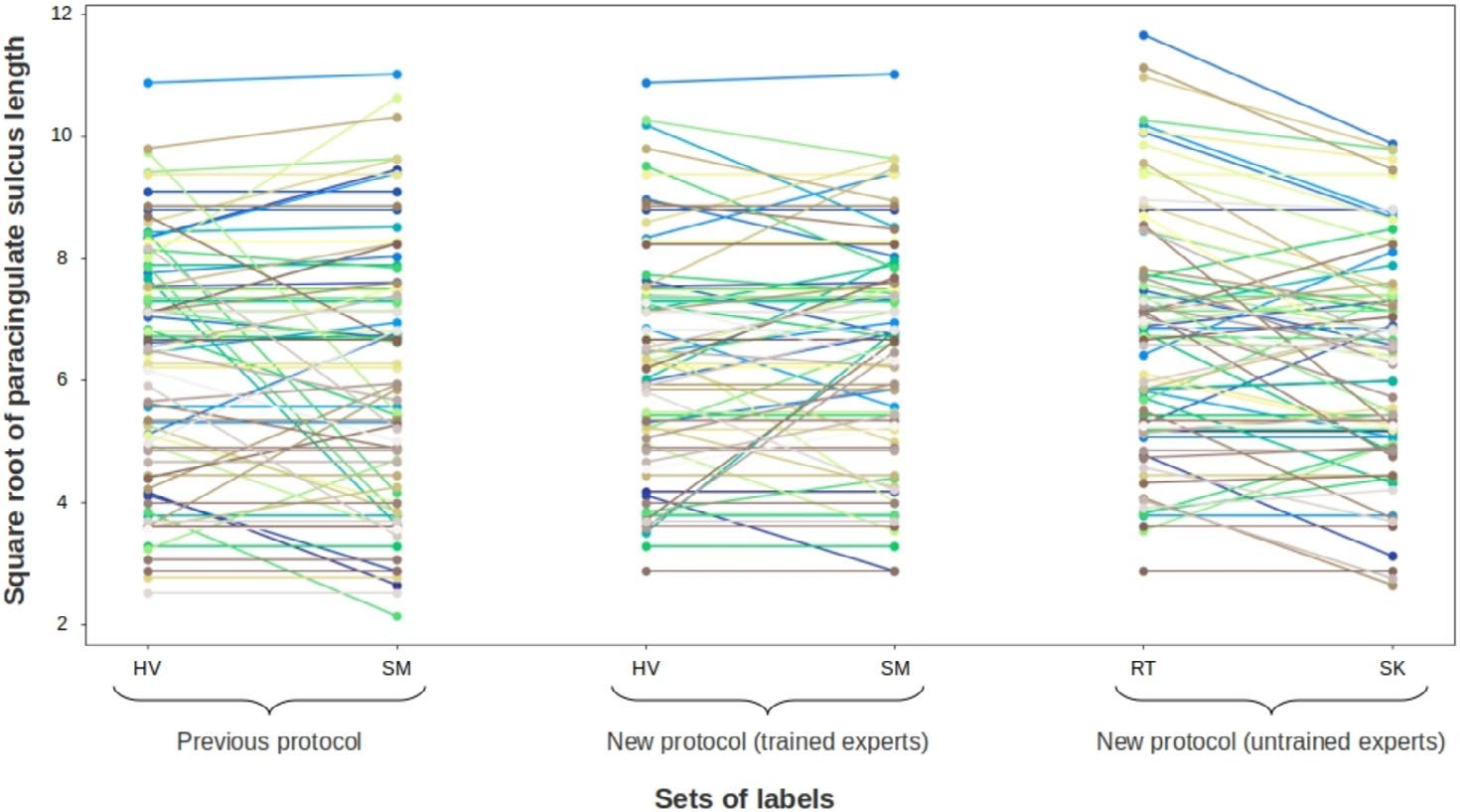
Pairwise plot of paracingulate sulcus length metric for pairs of raters. Pairwise plot of paracingulate sulcus length metric (square root of paracingulate sulcus length) for pairs of raters in the three different configurations: using the previous protocol or the new protocol, and with trained or untrained raters.

The results of the three sets of labels are presented on Figure 2. The lengths for left and right PCS identified by trained experimenters using the new protocol are presented in the Supporting Information S1.

After removing absent PCS measurements and applying a square‐root transformation to the remaining measurements, the resulting ICCs for ICCprevious, ICCnew and ICCnew_blind were 0.81, 0.85 and 0.86, respectively. All resulting ICCs would therefore be considered as good inter‐rater reliability (Koo and Li 2016), with *p*‐values < 0.001. The 95% confidence intervals were respectively: ICCprevious [0.72–0.87], ICCnew [0.78–0.90], ICCnew_blind [0.78–0.91]. These confidence intervals highlight that while the ICC scores show some slight increase, the gain in inter‐rater reliability with the new protocol is not statistically significant. These results reflect analysis combining data from both hemispheres; results from analysis of each hemisphere separately are shown in the Supporting Information S1 and are similar.

ICCs were additionally computed on the untransformed data, which also included absent PCS measurements (length = 0 cm). The resulting ICCs were 0.79 for ICCprevious, 0.90 for ICCnew and 0.87 for ICCnew_blind.

#### 3.1.2 Comparison of Resulting Length Metrics: Spearman Correlation

**FIGURE 3:**
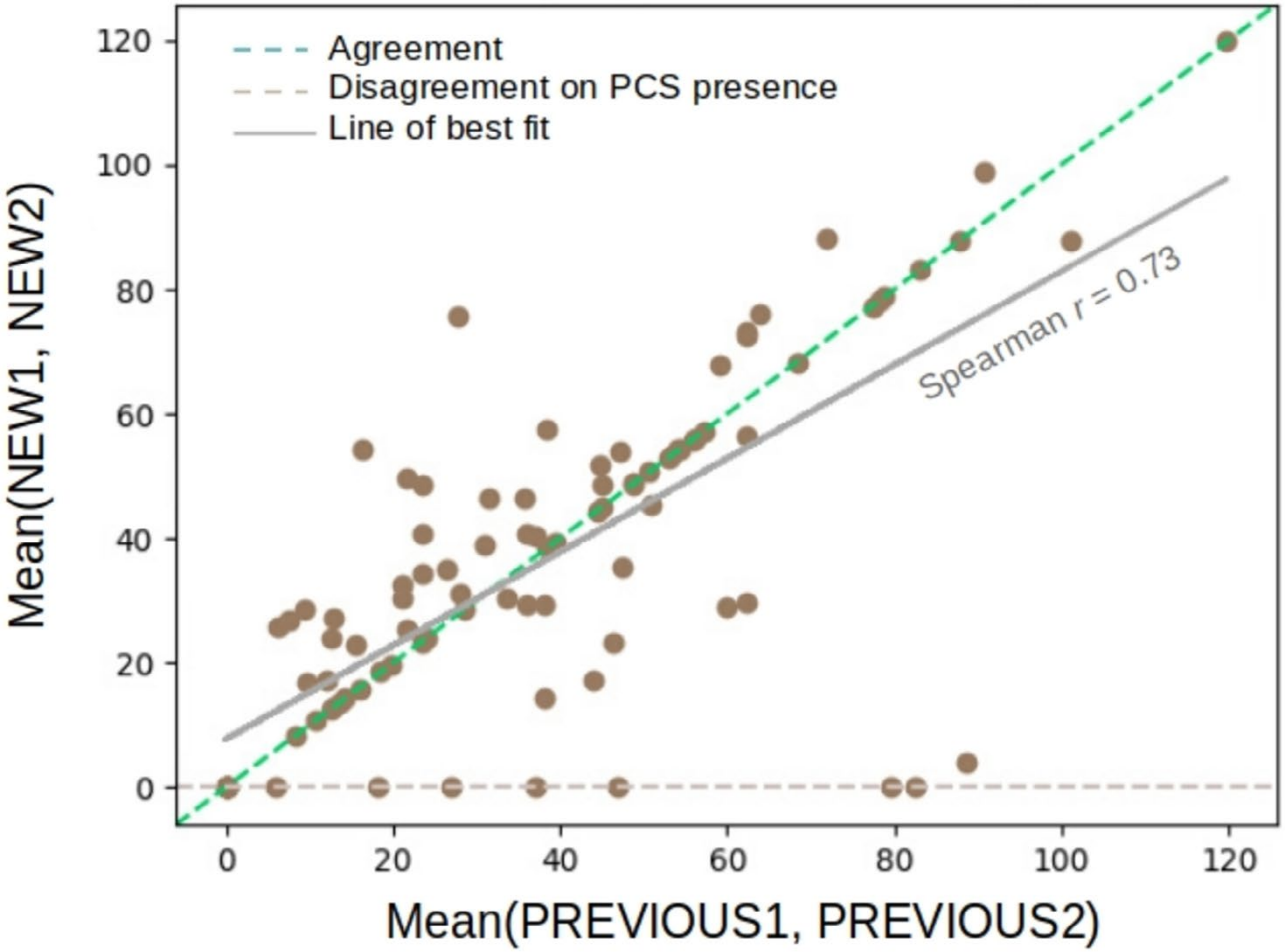
Scatter plot of the mean paracingulate sulcus (PCS) length obtained with the previous versus new protocol. Scatter plot of the mean PCS length obtained by HV and SM using the previous protocol (Garrison 2017) versus the new protocol (Mitchell et al. 2023), with a line of best fit (grey) and additional visual aids. The green dashed line represents where both protocols would show total agreement. The brown dashed line represents where there would be total disagreement on PCS presence.

The distribution of mean(NEW1, NEW2) compared to mean(PREVIOUS1, PREVIOUS2) is represented in Figure 3. Visual inspection of the scatter plot in Figure 3 highlights the presence of three distinct categories: agreement on PCS length, disagreement on PCS length, and disagreement on PCS presence (in 7 out of 100 hemispheres). From inspection of Figure 3, when both protocols identify a PCS but output different values for PCS length, the new protocol appears to output longer PCSs than the previous protocol, as expected due to the removal of the 20 mm rule. The disagreements on PCS presence all relate to occasions when the old protocol detects a PCS and the new protocol does not, which stem from the new protocol being able to label the intra‐limbic sulcus when present; in these cases, the old protocol effectively mislabels the intra‐limbic sulcus as cingulate, and hence then labels the cingulate as paracingulate. After removing the subjects that did not have a PCS according to at least one protocol, the resulting Spearman rank correlation was positive, with *r*(75) = 0.85, *p* < 0.001. Whilst recognising the qualitative aspect of different groups, we nevertheless calculate a Spearman rank correlation coefficient for all individuals combined as a summary indicator of methodological agreement; the resulting correlation was positive, with *r*(98) = 0.73, *p* < 0.001. Results from separate hemispheric analyses are in the Supporting Information S1, and are similar.

#### 3.1.3 Comparison of Resulting Length Metrics on Present PCS: Bland–Altman Method

**FIGURE 4:**
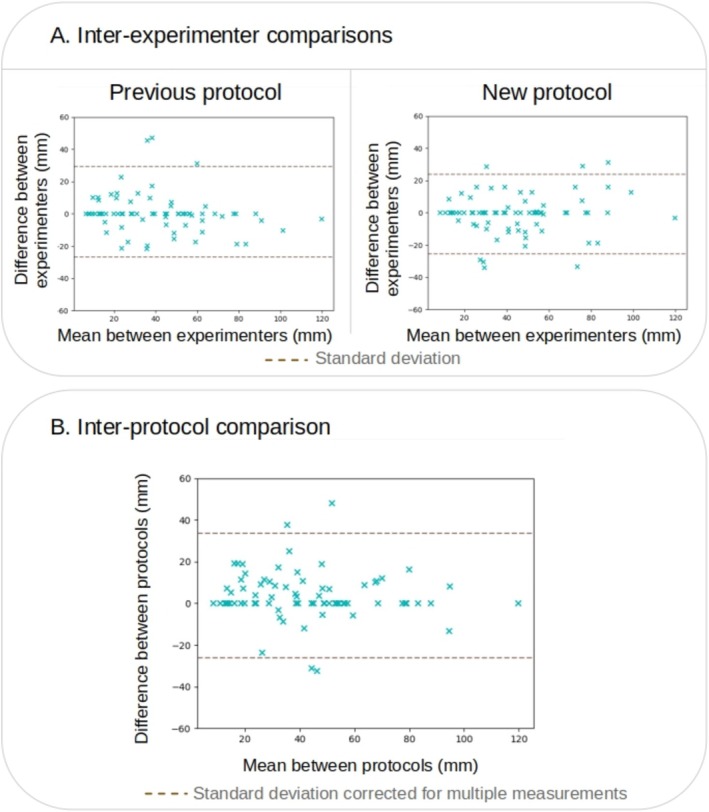
Scatter plots of difference versus mean using the Bland–Altman method. Scatter plots of difference versus mean for (A) inter‐rater comparison, using the previous (left) or new (right) protocols, and (B) inter‐protocol comparison. The data points are plotted in blue and the standard deviation as brown dashed lines. The differences between experimenters were computed as PREVIOUS1−PREVIOUS2. The differences between protocols were computed as mean(NEW)−mean(PREVIOUS).

After excluding subjects that have no PCS identified, the comparison of differences against mean PCS length are represented on Figure 4, for each protocol (Figure 4A) and between the means of both protocols (Figure 4B). The mean difference between experimenters HV and SM using the previous protocol was 1.31 mm, against −0.85 mm using the new protocol (the absolute mean difference was smaller using the new protocol). The standard deviation of differences using the previous protocol (14.01 mm) was higher than that using the new protocol (12.29 mm).

For the inter‐protocol comparison, the mean difference between protocols was 3.72 mm, and the standard deviation of differences corrected for repeated measurements was 14.97. Four out of 76 PCS lengths lie outside the limits of agreement defined as: mean difference ±2 standard deviations of differences (limits of agreement: −26 mm, +34 mm). Therefore, 95% of the PCS lengths will show a difference between both protocols comprised between −26 mm and 34 mm.

## 4 Discussion

This study is based on the development of a new protocol to identify the PCS. The agreement rates under the new and old protocols were assessed, as well as the relationship between the identification results obtained with the new protocol compared to the previous protocol.

Due to the important variability of sulcation in the medial prefrontal and cingulate cortices, the new protocol was developed to take advantage of 3D reconstructions of the brain, enabling improved sulcal identification. Since previous protocols identified sulci around the cingulate cortex based mostly on a single 2D‐slice (Yücel et al. 2001; Garrison 2017), some information relevant to sulcal identification was lost. Specifically, although the presence of the intra‐limbic sulcus is rare (present in 5% of cases, Paus, Tomaiuolo, et al. 1996), it can cause confusion and has been treated inconsistently in previous literature (Fornito et al. 2006). Since the cingulate sulcus is a primary fold (as reviewed in de Vareilles, Rivière, Mangin, and Dubois 2023), it is expected to be a deep landmark in the sulcal pattern. Therefore, in ambiguous cases, it can be helpful to use depth considerations to discriminate a possible intra‐limbic sulcus from a cingulate sulcus and a cingulate sulcus from a PCS. Taking this into account is facilitated using a 3D reconstruction and is introduced in the current protocol, allowing for more quantitative and objective decision making. From a practical point of view, using the BrainVISA software for sulcal identification automatically segments sulcal elements and allows users to copy‐paste sulcal labels to sulcal elements by just clicking on them, so it makes it quicker than having to manually delineate the sulcus using Mango, making it more practical for large‐scale datasets. Moreover, once the whole sulcal object is identified using the BrainVISA software, more automated metrics than length can be extracted (such as mean depth or opening, e.g., Mangin et al. 2020; Pizzagalli et al. 2020), or the whole 3D object for more refined shape considerations (e.g., Sun et al. 2016; de Vareilles et al. 2022; de Vareilles, Rivière, Pascucci, et al. 2023). The use of BrainVISA lays the foundation for future developments such as automated extraction of relevant features, such as number of PCS interruptions, which have been related to psychopathology (e.g., Meredith et al. 2012).

### 4.1 Methodological Decisions and Impact on Anatomical Objects

In addition to the depth considerations to differentiate a potential intra‐limbic sulcus from a cingulate sulcus, another methodological decision was made that departed from the previous protocol. While the seminal work considered the PCS as prominent if one or more elements formed a sulcus at least 20 mm long parallel to the cingulate sulcus (Paus, Tomaiuolo, et al. 1996), subsequent protocols have mostly accepted a partly arbitrary element of definition of the PCS sulcus, as exemplified in the “20 millimetres rule” of discarding sulcal elements matching the PCS when interruptions exceeded 20 mm (Yücel et al. 2001; Garrison 2017). While the PCS's extent is defined within a region constrained posteriorly by a marker defined based on the anterior and posterior commissures, in cases of PCS interruption previous protocols have defined the inclusion of sulcal elements within this region as dependent on the cumulative distance between all elements starting from the most anterior element. This leads to a situation in which two similar posterior sulcal elements could either be labelled as PCS or not depending on the distance to a more anterior sulcal element. In an extreme case, we can envision a situation where there would either be two distant PCS elements (one anterior and one posterior), or just the posterior one. In the dual configuration, the posterior element would be discarded, as it would be ‘too far from the beginning of the PCS’, while in the single configuration, the posterior element would be included as it would effectively be ‘the beginning of the PCS’ since no more anterior element is present. We consider this as an anatomical inconsistency as we posit the anatomical value of the posterior element to be the same in both cases (as argued in Leonard et al. 2009). Therefore, in the new protocol, sulcal elements are labelled as PCS based on their orientation and localisation in the brain but not on the overall distance of interruptions. We posit this adds consistency in the definition of the PCS, and effectively, it does not drastically affect the length of the PCS as these interrupted configurations (with more than 20 mm interruption) seldom present long posterior elements.

When looking into the mean PCS lengths obtained with both protocols, one can visually identify the major differences between both protocols. When the previous protocol identifies a PCS but the new protocol does not, it is often due to unusual folding ventral to the potential PCS. The new protocol would relate this folding to either the presence of an intra‐limbic sulcus or to a particularly interrupted cingulate sulcus, which would prevent the most dorsal part from being identified as a PCS, while the previous protocol would identify the most dorsal element as the PCS. An unusual folding ventral to the potential PCS can also lead to the new protocol identifying less PCS than the previous one if additional sulcal elements are located above the unusual folding. On the other hand, when the new protocol identifies a longer PCS than the previous one, it is due to the suppression of the “20 millimetres rule”, resulting in elements posterior to the interruptions but still within the first quadrant to be identified as PCS elements by the new protocol but not the previous one.

While anatomically relevant, both the accounting of the rare occurrence of an intra‐limbic sulcus and the removal of the 20 mm rule do not alter drastically the definition of the PCS, as demonstrated by the high correlation between the PCS lengths output by the two protocols.

When comparing the difference against the mean for identified PCSs in both protocols, the mean difference, as well as the standard deviation of the differences, showed some improvement using the new protocol compared to the previous one, with a mean difference closer to zero and a smaller standard deviation. When comparing the mean value obtained by both experimenters between the previous and new protocols, the mean difference is positive: the new protocol tends to identify longer PCSs on average. This implies that the removal of the “20 millimetres rule” has more impact on overall PCS length than the occasional disagreement between protocols over the identification of a cingulate sulcus versus PCS (which would systematically lead the previous protocol to identify a longer PCS than the new protocol). The limits of agreement show that in 95% of cases, the difference between both protocols is comprised between −26 mm and + 34 mm, which we consider an acceptable discrepancy given the overall span of PCS lengths (between 0 and 136 mm in the current cohort) and the modifications we voluntarily induced in the new protocol.

### 4.2 Reproducibility of New Protocol

Both protocols are shown to have high inter‐rater consistency (with all ICCs superior or equal to 0.79, whether including absent PCS or not). The new protocol also produced consistent results when used by independent untrained experts. While the ICCs on the whole cohort are higher using the new protocol, and the absolute mean difference and standard deviation of differences between HV and SM are smaller with the new protocol, no gain can be concluded on inter‐rater consistency using the new protocol compared to the previous one as the confidence intervals of the ICCs intersect. When assessing the left and right hemispheres separately (Supporting Information S1), the left hemisphere ICC under the previous protocol (after excluding absences) was 0.73, termed moderate inter‐rater reliability according to the classification of Koo and Li (2016), with the new protocol ICCs superior or equal to 0.84 (good reliability), but again the confidence intervals of the ICCs intersect.

The previous protocol was applied by the writers of the new protocol, which could induce bias. Nevertheless, it was demonstrated that the new protocol, which was developed to improve anatomical consistency in the identification of the PCS, showed high inter‐rater reliability, even when used by experimenters untrained on the protocol.

The protocol was assessed on a cohort of subjects with schizophrenia, schizophreniform, or schizoaffective psychosis. On such cohorts, it would be highly valuable to assess the sensitivity of sulcal features to variations within the schizophrenia continuum, though our sample sizes did not allow for such analyses. To go further, it would be very interesting to assess the reproducibility of the new protocol on other cohorts, notably on older cohorts with brain atrophy, to assess the reproducibility further on different kinds of sulcal variability.

### 4.3 Specifics of the BeneMin Cohort

The original BeneMin cohort comprised 74% male subjects and 26% female subjects. The subsample we have selected matches those proportions. Therefore, the subsample is not balanced in terms of gender. We argue the results are still relevant as a first proof of concept for the new protocol but we recognise that our sample is not representative of the general population.

Moreover, our dataset was comprised of individuals with psychiatric disorders (schizophrenia spectrum conditions) rather than a sample of people in perfect health, or a representative sample of the general population. It should not undermine the methodological assessment presented in this study, as there is some (although not conclusive) evidence schizophrenia may be associated with more interrupted cingulate and paracingulate sulci (Meredith et al. 2012), which would both make sulcal identification more ambiguous. Therefore, it is possible that studying a completely healthy cohort could result in an even higher ICC. It is conceivable that a range of lifestyle, demographic, and clinical factors may partly influence the reliability of PCS identification, but demonstrating reliability in a clinical setting is important for potential future application in applied research.

### 4.4 Conclusion

In conclusion, a new protocol is proposed for the identification of the paracingulate sulcus. This protocol offers high inter‐rater reliability by both experts and novel users alike, and we argue that it offers a more anatomically consistent definition of this variable brain landmark. We hope the new protocol will be useful in further investigations of the PCS, to reduce the uncertainty and ambiguity of PCS identification. Added to taking advantage of larger sample sizes where possible, we posit it would help arbitrate previous contradictory findings and bring to light anatomical correlates of cognitive and psychopathological features.

## Funding

The project was supported by Mental Health Research UK, Rosetrees Trust (A1841 and M864), and the MRC (MR/W020025/1). All research in the Department of Psychiatry at the University of Cambridge is supported by the National Institute for Health and Care Research (NIHR) Cambridge Biomedical Research Centre (NIHR203312) and the NIHR Applied Research Collaboration East of England.

## Supporting information


**Data S1:** hbm70574‐sup‐0001‐Supinfo.docx.

## Data Availability

The data that support the findings of this study are available on request from the corresponding author. The data are not publicly available due to privacy or ethical restrictions.
