# Supplementary material for: Evaluation of a New Paracingulate Sulcus Identification and Measurement Protocol
Source: Hum Brain Mapp. 2026 Jun 15;47(8):e70574. doi: 10.1002/hbm.70574 (PMC13266417; doi:10.1002/hbm.70574)
Supplement: Supplementary file 1 — Data S1: hbm70574‐sup‐0001‐Supinfo.docx. [file HBM-47-e70574-s001.docx]

**Supplementary Material:**

**Title:**

Evaluation of a New Paracingulate Sulcus Identification and Measurement Protocol

**Short running title:**

New PCS Measurement Protocol Evaluation

**Authors:**

Héloïse de Vareilles^1^ *, Samantha C. Mitchell^2^ *, Jane R. Garrison^2^, Shun-Chin Jim Wu^1^, Laura Alvarez-Sanchez^1^, Reuben Thomas^1^, Suveththa Kugan^1^, Atheer Al-Manea^1^, Michail Mamalakis^1,3^, Lynn Egeland Mørch-Johnsen^4,5^, Ingrid Agartz^6^, John Suckling^1^, Jon S. Simons^2^, Graham K. Murray^1,7^

**Affiliations:**

1. Department of Psychiatry, University of Cambridge, Cambridge, United Kingdom
2. Department of Psychology, University of Cambridge, Cambridge, United Kingdom
3. Department of Computer Sciences and Technology, University of Cambridge, Cambridge, United Kingdom
4. Norment, Division of Mental Health and Addiction, Oslo University Hospital, Institute of Clinical Medicine, University of Oslo, Oslo, Norway
5. Department of Psychiatry and Department of Clinical Research, Østfold Hospital, Grålum, Norway
6. Department of Psychiatric Research, Diakonhjemmet Hospital, Oslo, Norway
7. Cambridgeshire and Peterborough NHS Foundation Trust, Cambridgeshire, UK

** de Vareilles and Mitchell share first authorship*

Correspondence to: Graham Murray gm285@cam.ac.uk

**Index:**

1. **Paracingulate Sulcus Measurement Protocol V2. Page 2**
2. **Supplementary Results. Page 6**

**Paracingulate Sulcus Measurement Protocol**

**V2.**

**Tailored for BrainVISA**

Samantha C. Mitchell^1^*, Héloïse de Vareilles^2^*, Jane R. Garrison^1^, Atheer Al-Manea^2^,
John Suckling^2^, Graham K. Murray^2^, Jon S. Simons^1^

1. Department of Psychology, University of Cambridge
2. Department of Psychiatry, University of Cambridge

*Co-first authors

This protocol is an updated version of ‘Paracingulate Sulcus Measurement Protocol’ (Garrison, 2017 [https://doi.org/10.17863/CAM.9986](https://eur03.safelinks.protection.outlook.com/?url=https%3A%2F%2Fdoi.org%2F10.17863%2FCAM.9986&data=05%7C01%7Csm2596%40cam.ac.uk%7C9d23ab699e874cbb1caa08dbc0c106cb%7C49a50445bdfa4b79ade3547b4f3986e9%7C1%7C0%7C638315707495068567%7CUnknown%7CTWFpbGZsb3d8eyJWIjoiMC4wLjAwMDAiLCJQIjoiV2luMzIiLCJBTiI6Ik1haWwiLCJXVCI6Mn0%3D%7C3000%7C%7C%7C&sdata=sP3IW2CqLJi6k9%2By25Z9VkmsnKIhtpCfugJxqx%2BHIyU%3D&reserved=0)) and is now tailored for use in BrainVISA (<https://brainvisa.info>). The current protocol aims to identify and label the paracingulate sulcus using a 3D reconstruction of the grey-white matter interface of the brain and a visualisation of sulci as produced by the BrainVISA software. These reconstructions are dependent on the quality of the input T1 image and quality control of the raw T1 images (e.g., checking for movement artifacts, blur, or low grey/white contrast) is advisable prior to application of the protocol to the BrainVISA reconstructions.

| 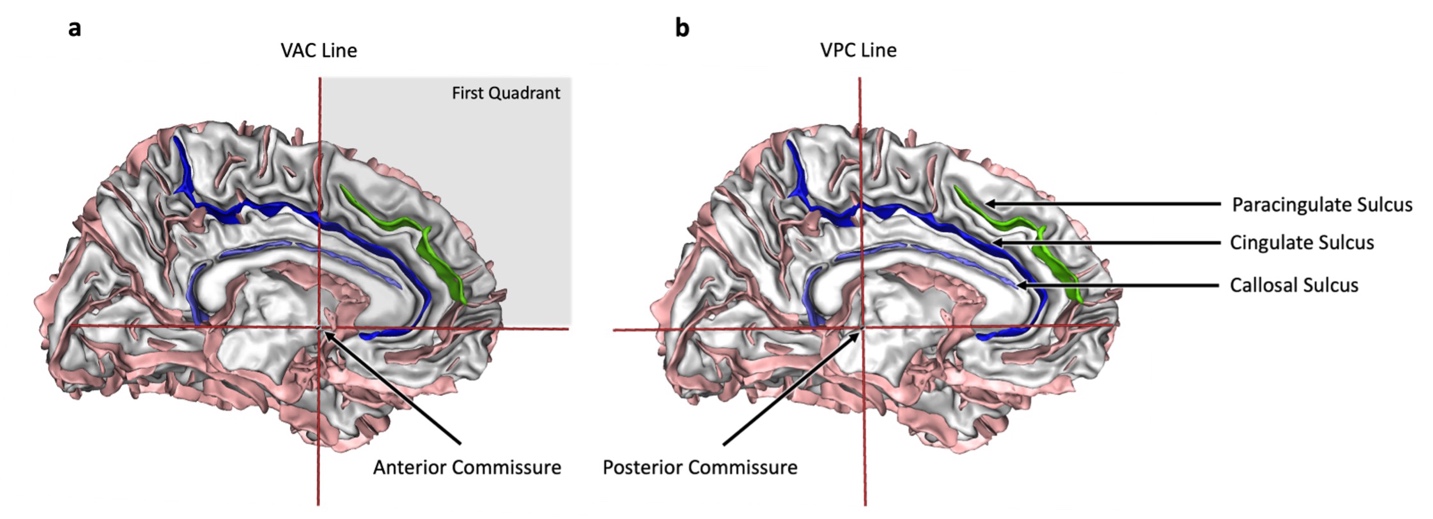**Vertical Anterior Commissure (VAC) and Vertical Posterior Commissure (VPC) lines:**  **Fig. 1. Representation of the VAC and VPC lines.** *The cross-mesh cursor is positioned on the anterior commissure (a) and posterior commissure (b), in a left hemisphere in which the callosal, cingulate and paracingulate sulci have been labelled.*  *In the ‘Talairach AC-PC referential’*   - When the cross hairs are placed on the anterior commissure, the vertical line is called the VAC line, and the upper anterior quadrant is hereby called the first quadrant (*cf.* Fig.1.a). - When the cross hairs are placed on the posterior commissure, the vertical line is called the VPC line (*cf.* Fig.1.b).   **BrainVISA cut tool:**   - The following protocol instructs the user to cut sulci when necessary. If the section to cut is too small, BrainVISA will not allow the cut to occur. In such cases, label the whole element according to its most dominant trait. |
| --- |

1. **Identify the callosal sulcus:** The callosal sulcus lies directly dorsal to the corpus callosum.

*(N.B. In some brains, this sulcus may be too shallow to be segmented by BrainVISA; identify its location based on indentations in the grey-white matter interface just over the corpus callosum).*

1. **Identify the cingulate sulcus:**

| 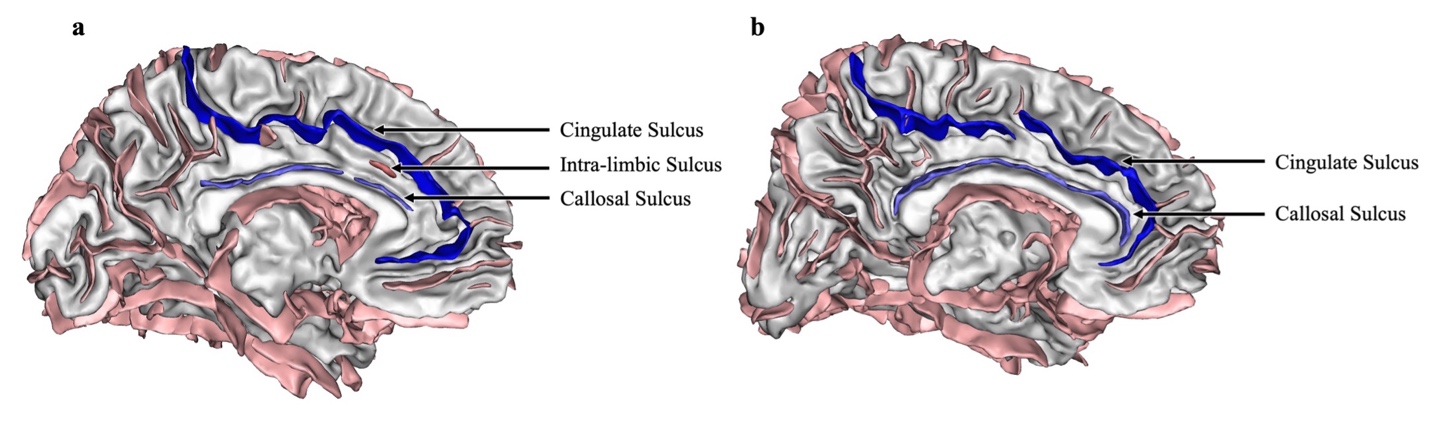***Fig 2. Examples of cingulate sulci (deep blue).*** *Continuous (a) and Interrupted (b) cingulate sulci, in left hemispheres in which the callosal and intra-limbic sulci have been labelled.*    The cingulate sulcus (Fig.2) is a primary sulcus and as such is expected to be deep and long. The posterior end of the cingulate sulcus is noticeably located posteriorly from the VPC line, behind the central sulcus, in the mid-sagittal section, with a ventral trajectory at first and curving to an anterior trajectory, predominantly parallel to the callosal sulcus. The cingulate sulcus extends throughout the first quadrant in most cases until at least the genu of the corpus callosum. The cingulate sulcus may be one continuous structure, or it may be interrupted (*i.e.,* split into smaller sections). |
| --- |

- 1. ***Identify the posterior cingulate sulcus element(s)****.* Starting posteriorly from the VPC line in the mid-sagittal section, with a ventral trajectory at first and curving to an anterior trajectory, predominantly parallel to the callosal sulcus. Label as cingulate sulcus.
  2. ***Identify the anterior cingulate sulcus element(s)*** – in continuity to the posterior cingulate sulcus.
     *N.B.* The cingulate can either be continuous or interrupted:
- *Continuous cingulate:* A continuous cingulate sulcus will run from behind the VPC line through to the genu of the corpus callosum (at least), remaining deep along its path.
- *Interrupted cingulate:* An interrupted cingulate will be composed of separate sulcal elements which together form a sulcal structure resembling a continuous cingulate. Note that merging of sulci including both cingulate and non-cingulate elements can happen and can be identified by noticeable drops in depth along the resulting sulcal combination.

Guidelines:

- *Depth:* Cingulate sulcal elements should be at least half as deep as the deepest sulcus from the first quadrant. If there are noticeable drops in depth within a cingulate sulcal element, consider it as split into two sulci. Consider that the cingulate may have merged either with another cingulate element or with a non-cingulate element, based on the trajectory and depth criteria. If relevant, use the BrainVISA cut tool to separate the elements for accurate labelling.
- *Trajectory:* Once the depth rule is applied, if there is remaining uncertainty, use the trajectory of the sulcal elements to aid the decision. The anterior elements of the cingulate sulcus should globally follow the trajectory of the most ventral part of the posterior cingulate sulcal element (close to the VAC line) and remain predominantly parallel to the callosal sulcus.
- *Vertical Branches:* Vertical branches stemming from the cingulate sulcus/sulcal elements should not be labelled.

1. **Identify whether there are intra-limbic sulci:** If there are sulcal elements located between the cingulate sulcus and the callosal sulcus, label them as intra-limbic sulci (*cf.* Fig.2.a)

1. **Identify whether there is a paracingulate sulcus:**

| 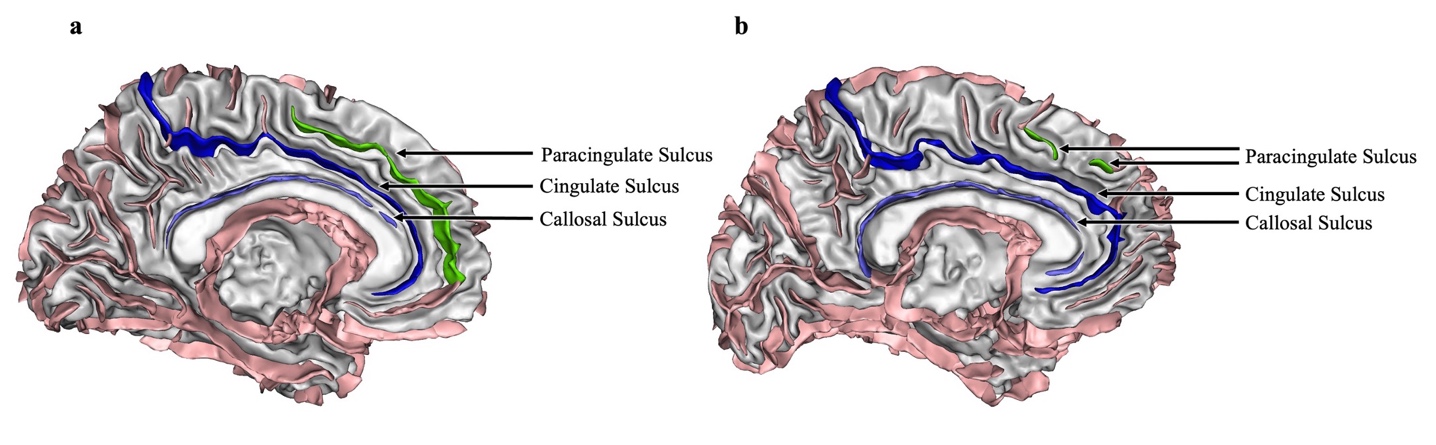***Fig 3****.* ***Examples of paracingulate sulci (green)****. Continuous (a) and interrupted (b) paracingulate sulci in left hemispheres in which the callosal and cingulate sulci have been labelled.*  The paracingulate sulcus (Fig.3) is located directly dorsal and parallel to the cingulate sulcus and is oriented with a posterior trajectory. The paracingulate sulcus cannot be present in the absence of a cingulate sulcus. Sometimes the paracingulate sulcus is clear, other times it is completely absent, but it is usually there in part and often interrupted/broken. The paracingulate sulcus/sulcal elements should always move in a posterior direction, towards the back of the brain, and be predominantly parallel to the cingulate sulcus and essentially horizontal. It can help to imagine a vertical line from the horizontal crosshair, and the paracingulate sulcus should move away from this towards the back of the brain. |
| --- |

Start labelling the paracingulate sulcus (or sulcal elements) from the point in the first quadrant where the paracingulate sulcus first moves in a posterior direction. Stop labelling at the VAC line, except if the sulcus, or a sulcal element, continuously flows posteriorly beyond the VAC line – in this case, stop labelling at the point where the sulcus is no longer parallel to the cingulate sulcus and stops having a posterior trajectory. Do not include any elements that start posterior to the VAC line.

Guidelines:

- *Inclusion:* Do not include any sulcal elements that are vertical or do not have a posterior trajectory unless these elements are parallel to the cingulate sulcus.
- *Orientation:* In case of doubt on orientation, rely on the orientation of the indentations in the grey/white matter interface embedding the sulcal element.
- *Branches:* Do not include any branches stemming out from the paracingulate sulcus/sulcal elements.
- *Change in trajectory:* In case of change of trajectory within a sulcal element, cut it and only label the section with the posterior trajectory.

*N.B. in cases of uncertainty when labelling any sulci, reference to the raw T1 image may be helpful*

| ***Key changes from Garrison, 2017***     - The original protocol specified that the total distance between paracingulate sulcus elements should be no more than 2cm. This rule has been removed, as it resulted in visually analogous sulcal elements being labelled differently depending on their spacing with anterior paracingulate sulcus elements. As a result, i) previously discarded elements are now included in the paracingulate sulcus, and ii) the paracingulate sulcus’s configuration may now be sparse. - The current protocol guides the reader through labelling sulci in the cingulate region in a specified order to improve the accuracy and consistency of paracingulate sulcus identification. - The current protocol acknowledges the potential presence of intra-limbic sulci. - As the current protocol utilises 3D visualisations of sulci, it allows the reader to use depth as a criterion for cingulate sulcus identification. |
| --- |

Garrison, J. (2017). Paracingulate Sulcus Measurement Protocol. Apollo - University of Cambridge Repository. <https://doi.org/10.17863/CAM.9986>

**Supplementary results**

**Left versus right PCS lengths resulting from trained experts using the new protocol**
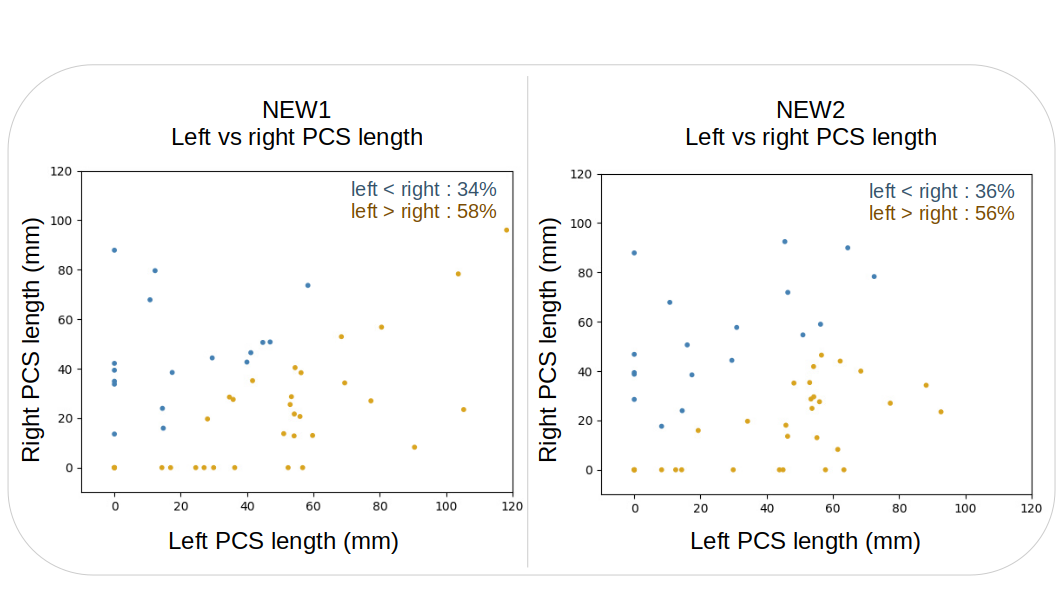
Scatterplot of left versus right PCS lengths of 50 subjects obtained after identification by two different trained experts using the new protocol. Note that 4 subjects had no PCS on either hemisphere. NEW1 and NEW2 are results from the two experts HV and SM using the new protocol.

**Hemisphere-specific ICCs**

*Left hemisphere:*

After removing absent PCS measurements and applying a square-root transformation to the remaining measurements, the resulting ICCs for ICCprevious, ICCnew and ICCnew_blind on the left hemisphere were 0.73, 0.85 and 0.84, respectively, with p-values < 0.001. According to the recommendations of Koo and Li (Koo and Li, 2016), ICCprevious would be considered as having moderate inter-rater reliability, and ICCnew and ICCnew_blind as having good inter-rater reliability. The 95% confidence intervals were respectively: ICCprevious [0.55 - 0.84], ICCnew [0.73 - 0.92], ICCnew_blind [0.72 - 0.92].

*Right hemisphere:*

After removing absent PCS measurements and applying a square-root transformation to the remaining measurements, the resulting ICCs for ICCprevious, ICCnew and ICCnew_blind on the right hemisphere were 0.87, 0.85 and 0.87, respectively. All resulting ICCs would therefore be considered as good inter-rater reliability (Koo and Li, 2016), with p-values < 0.001. The 95% confidence intervals were respectively: ICCprevious [0.76 - 0.93], ICCnew [0.73 - 0.92], ICCnew_blind [0.78 - 0.93].

*Length metrics of the two protocols: Spearman correlation restricted to non-zero measures*

Left: *r*(38) = 0.85, *p* < 0.001

Right: *r*(38) = 0.82, *p* < 0.001.

Whilst recognising the qualitative aspect of different groups, we nevertheless also calculate Spearman rank correlation coefficients for all individuals combined as a summary indicator of methodological agreement:

Left *r*(50) = 0.62, *p* < 0.001.

Right r(50) = 0.82 p < 0.001

*Comparison of length metrics on present PCS: Bland-Altman method by hemisphere*

Left:

The mean difference between experimenters HV and SM using the previous protocol was 4.09mm, against 1.08 mm using the new protocol. The standard deviation of differences using the previous protocol (16.21mm) was higher than that using the new protocol (12.80mm).

For the inter-protocol comparison, the mean difference between protocols was 1.71 mm, and the standard deviation of differences was 15.08 (corrected).

Uncorrected Limits of Agreement: [-20.25, 23.68]

Corrected Limits of Agreement: [-28.44, 31.87]

Right:

The mean difference between experimenters HV and SM using the previous protocol was –1.47mm, against -2.79 mm using the new protocol. The standard deviation of differences using the previous protocol (10.69mm) was lower than that using the new protocol (11.44mm).

For the inter-protocol comparison, the mean difference between protocols was 5.74mm, and the standard deviation of differences corrected for repeated measurements was 14.38

Uncorrected Limits of Agreement: [-18.39,29.86]

Corrected Limits of Agreement: [-23.02,34.50]

Scatter plots, by hemisphere, of difference versus mean for inter-rater comparison, using the previous or new protocols, and inter-protocol comparison. Hemispheres with a zero rating from either experimenter under either protocol have been excluded from the analysis. The data points are plotted in blue and the standard deviation as brown dashed lines for all plots; the solid brown line is the mean. The differences between experimenters were computed as PREVIOUS1 - PREVIOUS2. The differences between protocols were computed as mean(NEW) - mean(PREVIOUS).


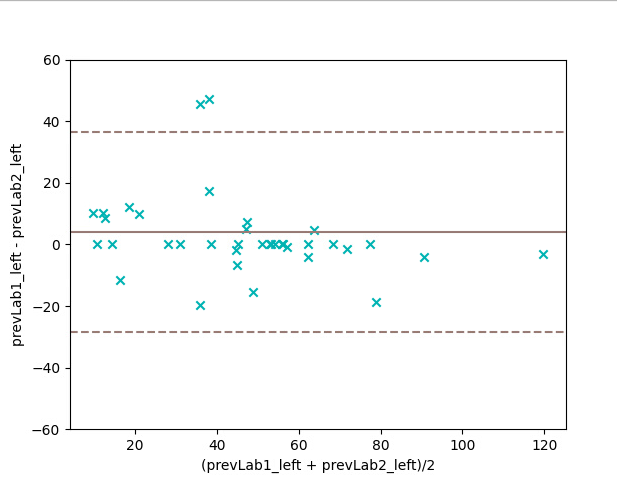


Inter-experimenter comparison of the previous protocol for the left hemisphere, with mean of experimenters on the x-axis (mm) and difference between the experimenters on the y-axis (mm).


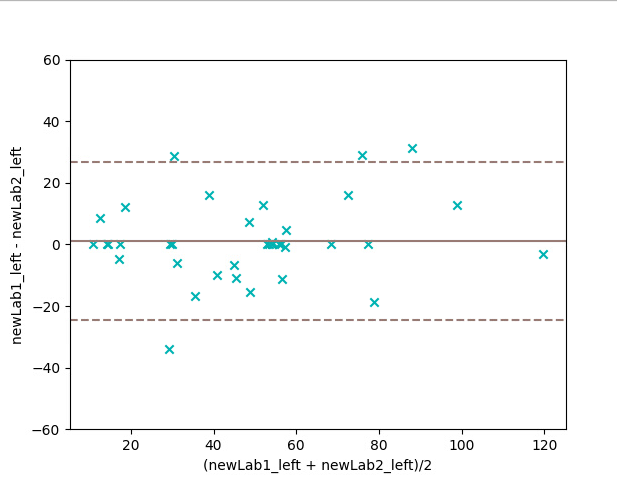


Inter-experimenter comparison of the new protocol for the left hemisphere, with mean of experimenters on the x-axis (mm) and difference between the experimenters on the y-axis (mm).


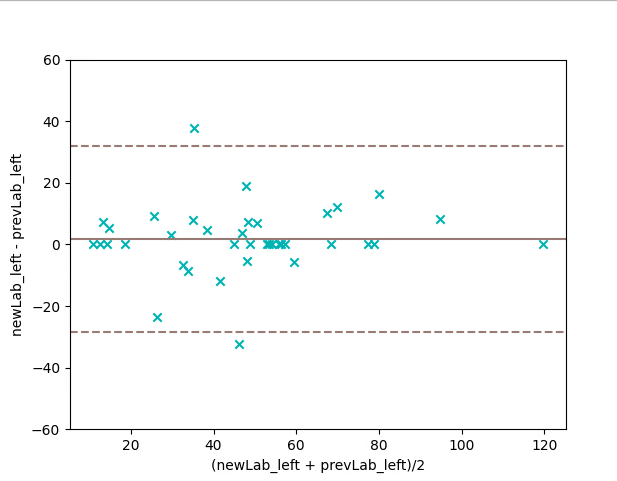


Inter-protocol comparison for the left hemisphere, with mean of protocols on the x-axis (mm) and difference between the protocols on the y-axis (mm).


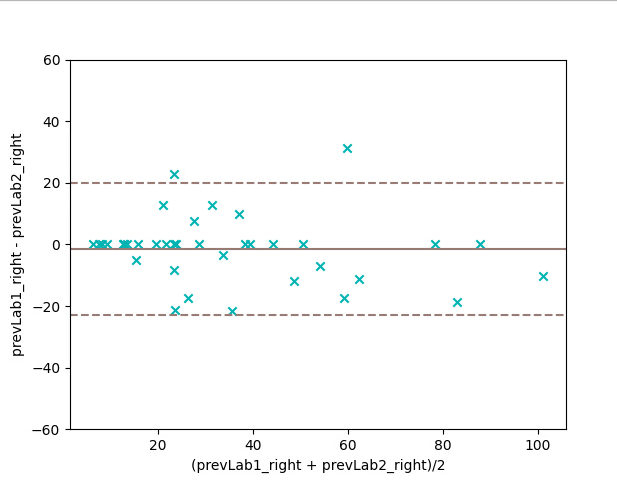


Inter-experimenter comparison of the previous protocol for the right hemisphere, with mean of experimenters on the x-axis (mm) and difference between the experimenters on the y-axis (mm).


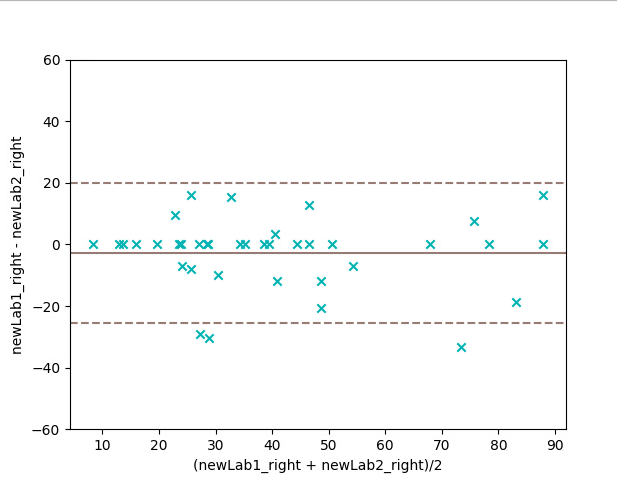


Inter-experimenter comparison of the new protocol for the right hemisphere, with mean of experimenters on the x-axis (mm) and difference between the experimenters on the y-axis (mm).


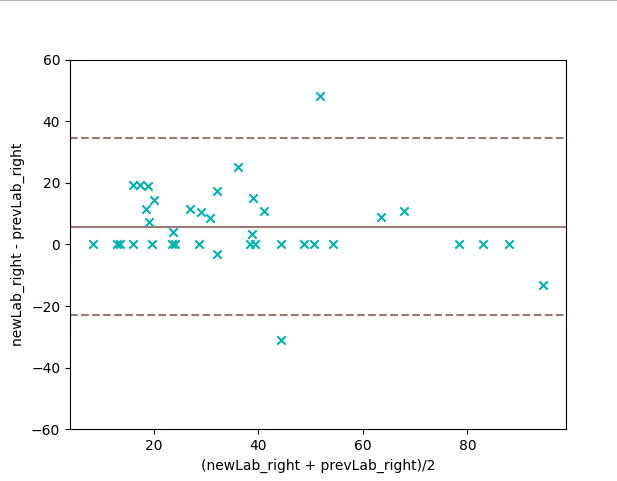


Inter-protocol comparison for the right hemisphere, with mean of protocols on the x-axis (mm) and difference between the protocols on the y-axis (mm).

Koo, T.K., Li, M.Y., 2016. A Guideline of Selecting and Reporting Intraclass Correlation Coefficients for Reliability Research. Journal of Chiropractic Medicine 15, 155–163. <https://doi.org/10.1016/j.jcm.2016.02.012>
